# Atomic electric fields revealed by a quantum mechanical approach to electron picodiffraction

**DOI:** 10.1038/ncomms6653

**Published:** 2014-12-15

**Authors:** Knut Müller, Florian F. Krause, Armand Béché, Marco Schowalter, Vincent Galioit, Stefan Löffler, Johan Verbeeck, Josef Zweck, Peter Schattschneider, Andreas Rosenauer

**Affiliations:** 1 Institut für Festkörperphysik, Universität Bremen, Otto-Hahn-Allee 1, 28359 Bremen, Germany; 2 Center of Excellence for Materials and Processes, Universität Bremen, Otto-Hahn-Allee 1, 28359 Bremen, Germany; 3 EMAT, University of Antwerp, Groenenborgerlaan 171, B-2020 Antwerp, Belgium; 4 Institut für Experimentelle und Angewandte Physik, Universität Regensburg, Universitätsstraße 31, 93040 Regensburg, Germany; 5 Institute of Solid State Physics, Vienna University of Technology, Wiedner Hauptstraße 8-10/E138, A-1040 Vienna, Austria; 6 University Service Centre for Transmission Electron Microscopy, Wiedner Hauptstraße 8-10/E052, A-1040 Vienna, Austria

## Abstract

By focusing electrons on probes with a diameter of 50 pm, aberration-corrected scanning transmission electron microscopy (STEM) is currently crossing the border to probing subatomic details. A major challenge is the measurement of atomic electric fields using differential phase contrast (DPC) microscopy, traditionally exploiting the concept of a field-induced shift of diffraction patterns. Here we present a simplified quantum theoretical interpretation of DPC. This enables us to calculate the momentum transferred to the STEM probe from diffracted intensities recorded on a pixel array instead of conventional segmented bright-field detectors. The methodical development yielding atomic electric field, charge and electron density is performed using simulations for binary GaN as an ideal model system. We then present a detailed experimental study of SrTiO_3_ yielding atomic electric fields, validated by comprehensive simulations. With this interpretation and upgraded instrumentation, STEM is capable of quantifying atomic electric fields and high-contrast imaging of light atoms.

## Abstract (web summary)

Advances in electron microscopy are enabling ever smaller features to be probed, with the measurement of atomic electric fields standing as a major challenge. Towards that aim, Müller *et al.* present a simplified theoretical approach for enhancing the resolution in differential phase contrast microscopy.

## Graphical abstract

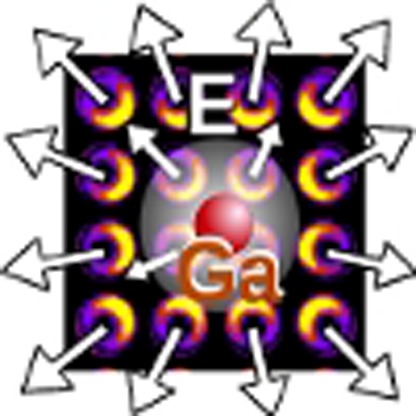

Innovative nanoelectronic devices such as ferroelectric tunnel junctions^1^,^2^ in the field of nonvolatile memory technology, or gallium nitride (GaN)-based^3^,^4^,^5^ laser diodes in optoelectronic applications rely on electric properties at the atomic scale. In both fields, differential phase contrast (DPC) experiments^6^,^7^,^8^ are considered as a key for the understanding of tunnelling electroresistance^9^ and the quantum-confined Stark effect^10^, respectively. On the basis of its success in the quantification of magnetic fields^11^,^12^,^13^ varying on the micrometre scale, conventional DPC is predicted to be able to map atomic electric fields at the picometre scale by aberration-corrected scanning transmission electron microscopy (STEM), and prospects to detect electron redistributions due to chemical bonding have been given^6^. Recently, impressive DPC experiments have been performed in which signatures of atomic electric fields, including ionicity, have been observed^8^ by recording portions of diffraction patterns on a segmented annular bright-field detector^14^,^15^,^16^. The design of this detector is derived from the assumptions that the central part of the diffraction pattern (the ‘ronchigram’) consists of a homogeneously filled disc, and that the Lorentz force causes a constant deflection of the electron beam as a whole.

In retrospect on nearly 100 years of electron diffraction in which elaborate theories have been developed to describe the complexness of multiple scattering of relativistic electrons in crystals^17^,^18^, this view on DPC impresses by its striking simplicity. In particular, electron ronchigrams rather appear as an artwork created by the sensitive interplay of scattering geometry, specimen thickness, probe parameters and crystal potential than simply being a shifted copy of a homogeneously illuminated disc. It is thus desirable to enhance DPC microscopy such that it can take intensity variations in diffraction patterns into account, and relate them quantitatively to atomic electric fields. In particular, this would be an important step as to studying electronic properties in nanotechnology with aberration-corrected STEM since Maxwell’s equations, furthermore, allow for the conversion of electric fields to charge- and electron densities.

In this work, we first lay the theoretical basis for the measurement of electric fields independently of the complexness of ronchigrams by exploiting the axioms of quantum theory and Ehrenfest’s theorem. By assuming that propagation and scattering of the STEM probe are negligible in thin specimen, we can relate the quantum mechanical expectation value for the momentum transfer to the electric field convolved with the probe intensity. Second, we develop the full method in a simulation study of GaN to show the validity of this assumption and demonstrate the possibility of mapping atomic electric fields, charge- and electron densities using a crystal where especially the latter exhibit a simple configuration. Finally, we present the electric field determined experimentally in SrTiO_3_. This is accompanied by comprehensive simulations accounting for the experimental conditions to validate the SrTiO_3_ results also theoretically. As our quantum mechanical interpretation formally results in a centre of mass calculation to measure the expectation value for the momentum transfer, we conclude that high-resolution DPC can be enhanced significantly by using two-dimensional (2D) pixel arrays instead of conventional segmented detectors.

## Results

### A quantum mechanical approach to DPC

**Figure 1:**
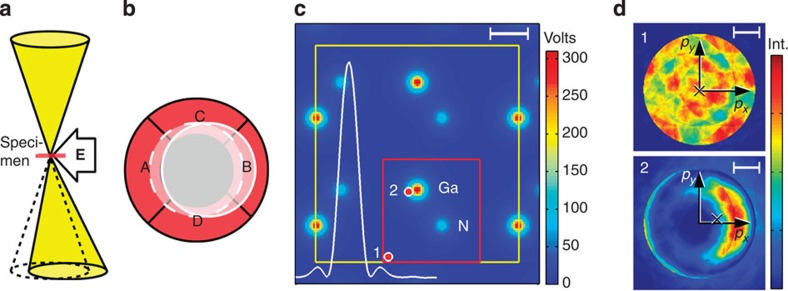
Established view on STEM DPC experiments in contrast to typical data for GaN. (**a**) The electron beam is focused on a thin specimen. Conventionally, an electric field **E** is thought to cause an angular deflection with respect to **E**=0 (dashed). (**b**) Detector used in conventional DPC to detect the shift of the central disc in the diffraction pattern (ronchigram). Given a homogeneously filled disc, this shift is determined by intensity differences between opposite ring segments *A*–*B* and *C*–*D*. By calibration, this signal is converted to an angular deflection and to electric/magnetic fields finally. (**c**) Coulomb potential of a GaN crystal obtained by DFT 21, projected along [
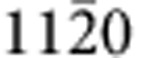
]. The white line shows the intensity profile of the incident electron beam. The yellow region marks a GaN unit cell. Scale bar length is 100 pm. Diffraction patterns have been simulated for 80 × 80 probe positions in the red region. Markers 1 and 2 refer to the probe positions for the diffraction patterns in **d**. Ronchigrams simulated at position 1 (top, nearly no electric field) and position 2 (bottom, high electric field varying across the probe). The ronchigram is neither shifted at non-zero field nor does it exhibit homogeneous intensity. Integration according to equation (1) yields the quantum mechanical momentum transfer 
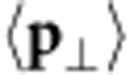
 drawn as crosses. Length of the white scale bar is 10 mrad. Momentum space axes *p*_*x*,*y*_ have a length of 10*h* nm^−1^.

We contrast the established view on DPC with typical STEM data in Fig. 1. As illustrated by the set-up of conventional DPC experiments in Fig. 1a,b, a non-zero electric field is supposed to cause a global angular deflection of the STEM probe, leading to a characteristic shift of the ronchigram. This translates into a difference signal in opposite detector segments A and B. On the basis of the wave optical picture in Fig. 1c with an airy-shaped profile of a contemporary aberration-corrected probe (white) we raise the question whether the classical picture of Fig. 1a,b is realistic for the investigation of the underlying Coulomb potential of GaN (colour-coded). Exemplarily, the ronchigrams in Fig. 1d have been simulated for the STEM probe at positions labelled 1 and 2 in Fig. 1c. Even at the interstitial position 1 a rich inner structure is observed. The bottom ronchigram in Fig. 1d simulated for an electron probe focused near a gallium site exhibits no shift but a complex intensity transfer to the right, predominantly in the interior of the ronchigram. This explains why a segmented detector as in Fig. 1b is suitable to obtain high contrast at atomic sites^8^ whereby the quantification of electric fields in terms of ronchigram shifts can be inaccurate.

We solve this problem by looking at diffraction patterns from the standpoint of basic quantum mechanics. As specimen and Fraunhofer diffraction plane are related by Fourier transform 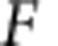, it is instructive to recall that 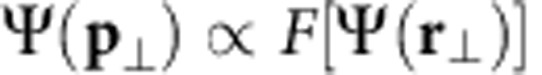 is an equivalent representation of the object exit wave function Ψ with 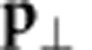 and 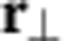 momentum and real space coordinates perpendicular to the incident electron beam, respectively. By the axioms of quantum mechanics, eigenvalues 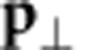 of the momentum operator are observable with probability 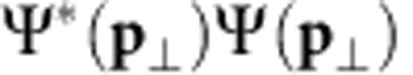. This is the normalized intensity distribution 
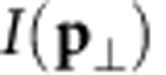
 in the diffraction plane for a sufficiently large number of detected electrons. Furthermore, the quantum mechanical expectation value for the momentum is


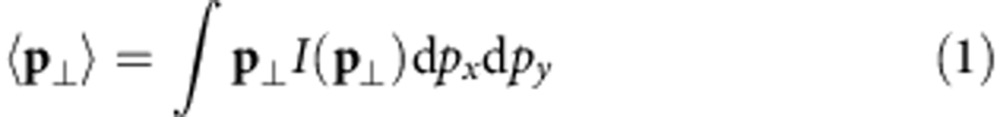


which equals the average momentum determined from repeated single-electron experiments for which 
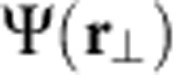
 is a solution to the Schrödinger equation. Note that we assume 
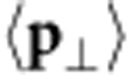
=0 for the incident probe so that 
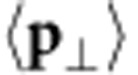
 is equivalent to the expectation value for the momentum transfer caused by interaction with the specimen. Equation (1) condenses the rich structure of the diffraction pattern into the average momentum 
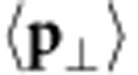
, which is necessary and sufficient for our aim, as will be shown below. In order to obtain 
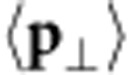
 experimentally, a detector should be capable of performing the respective summation, for example, by using CCDs (charge-coupled devices). As equation (1) equals a centre of mass calculation formally, it becomes obvious that conventional DPC detectors as in Fig. 1b can fail to deliver 
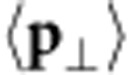
 from the moment the ronchigram exhibits inhomogeneous intensity.

The diffraction patterns in Fig. 1d include momentum transfers calculated from equation (1) as crosses. This yields 
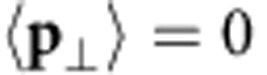
 and 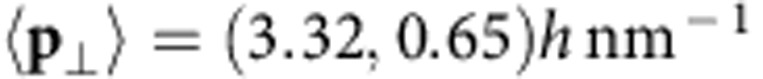 for positions 1 and 2, respectively, with Planck’s constant *h*=6.6261 × 10^−34 ^Js. Unlike the conventional DPC technique in Fig. 1b, this quantitatively exploits the detailed intensity redistributions caused by the electric field.

The Lorentz force is equal to momentum transfer per time in classical electrodynamics. According to Ehrenfest’s theorem^19^, this still holds in quantum mechanics for the expectation value 
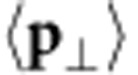
 of the momentum transfer for the case considered here. Electrons with velocity *v* spend the time *z*/*v* in a specimen of thickness *z* from which we can derive the following simplified relation between momentum transfer and measured electric field 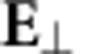:


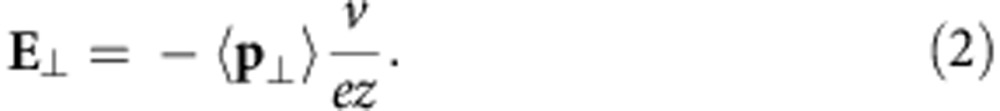


Here, *e*=1.6022 × 10^−19 ^C is the elementary charge and 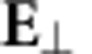 the projection average along the electron beam direction. We note here that the derivation of equation (2) formally involves the integration over the full three-dimensional (3D) electric field in the specimen, weighted with the local intensity of the electron beam. The latter is not known in experiment and changes as the electron wave passes the specimen due to scattering and propagation. To derive equation (2), we therefore simplified this problem by assuming that the intensity of the STEM probe does not change along the *z* direction. Hence, equation (2) is only valid for small specimen thicknesses (1–2 nm) where a broadening of the electron beam can be neglected (Supplementary Note 1 and Supplementary Figure 1). As the propagation of the electron wave is, furthermore, affected by a curvature of the phase of the incident wave, careful adjustment of aberrations and focus is required for the method. Finally, the measured electric field 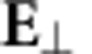 is a convolution of the true electric field 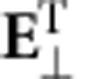 with the normalized probe intensity *I*_Probe_,


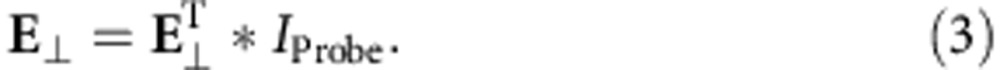


For the detailed derivation of equations (1, 2, 3) we refer to Supplementary Note 2.

### Methodical study using GaN

The power of this concept is initially demonstrated in a theoretical case study of GaN in 
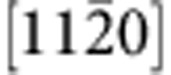
 projection. Here atomic columns are each occupied by a single atomic species with a comparably high difference in atomic number (*Z*_Ga_=31, *Z*_N_=7), making it suitable to study large and small electric fields at respective sites simultaneously. Contrary to SrTiO_3_ chosen in the experiment below because of the large separation of 195 pm of adjacent columns, GaN exhibits a very clear electronic configuration, which is attractive in view of the derivation of charge- and electron densities.

**Figure 2:**
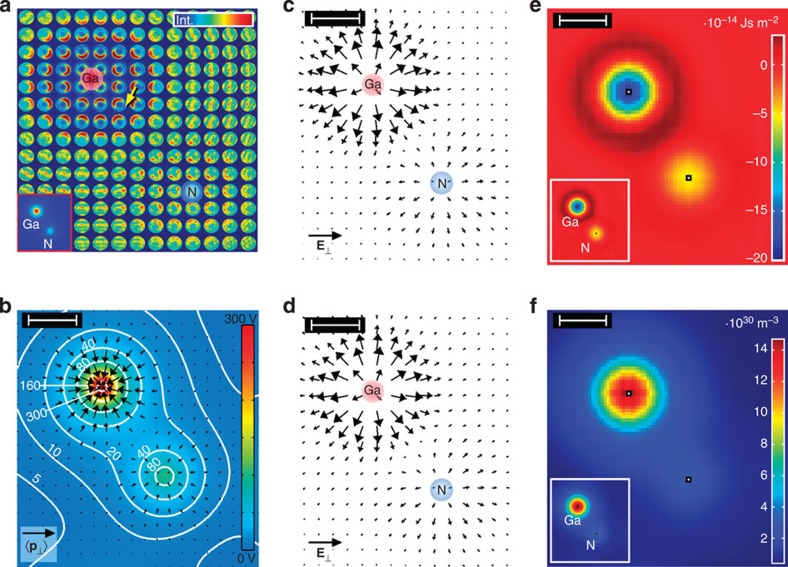
Simulations for the methodical study of GaN. (**a**) Ronchigrams of GaN simulated for 1.3 nm specimen thickness, arrayed with respect to the position of the STEM probe. The primary effect of the atomic electric field is a redistribution of intensity (Int., colour-coded) within the ronchigrams. The arrow denotes intensity transferred even to opposite direction. All data in this figure are based on these simulations. For better visibility, only subsets of the 80 × 80 raster are shown in **a**–**d**. (**b**) Vector field for the expectation value of the momentum transfer calculated from equation (1) and the ronchigrams in **a**. The momentum transfer correlates directly with the gradient of the Coulomb potential shown colour-coded and as white isolines (Volt units). Length of the black legend vector is 5*h* nm^−1^. (**c**) Electric field 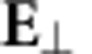 derived from the momentum transfer in **b** via equation (2), showing the radial characteristic at both sites. The field strength decreases near nuclei because of the extension of the STEM probe. Length of the black legend vector is 3 V pm^−1^. (**d**) Electric field used in the simulation (negative gradient of potential in **b**), convolved with the probe intensity according to equation (3). It agrees nearly perfectly with part **c** with a maximum error of 0.1 V pm^−1^. Length of the black legend vector is 3 V pm^−1^. (**e**) Divergence of the momentum transfer in **b**, being proportional to the charge density according to equation (4). The inset depicts the theoretical result obtained by DFT. (**f**) Electron density calculated from the divergence of the momentum transfer in **b** according to equation (4) with the proton density entering as prior knowledge. The inset shows the electron density obtained directly by DFT, convolved with the probe intensity. Scale bars top left in **b**–**f** are 50 pm.

Multislice^17^ simulations^20^ have been carried out at 80 × 80 scan points in the red region of Fig. 1c (see Methods section for details). Ronchigrams at 1.3 nm specimen thickness have been arrayed with respect to the probe positions in Fig. 2a. Atomic sites become evident from the redistribution of intensities inside the ronchigrams that reflects the tendency of electrons to channel along atomic columns. This effect is enhanced near gallium because of a more-than-four times larger nuclear charge compared with nitrogen. If we look at ronchigrams near gallium more closely, we see that intensity is partly also transferred in the opposite direction to form a diffuse halo as indicated in yellow in Fig. 2a. This can be explained by the finite extension of the STEM probe according to Fig. 1c because different parts of the probe experience different electric fields.

However, regardless of the complex intensity patterns inside the ronchigrams, we can rely on equation (1). Performing this integration yields the vector field for the momentum transfer 
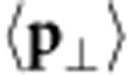
 drawn in Fig. 2b, superimposed to the colour-coded Coulomb potential with selected isolines. With Coulomb’s law and equation (2) in mind, this vector field is intuitively satisfying because the momentum transfer is invariably perpendicular to equipotential lines, is antiparallel to the electric field and increases drastically in the vicinity of atomic sites. However, the maximum momentum transfer of 4*h* nm^−1^ occurs 30 pm away from the gallium site and decreases again towards the nucleus, which is expected because of the finite probe size as a result of equation (3).

By virtue of equation (2), the electric field 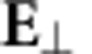 has been calculated from Fig. 2b and is depicted in Fig. 2c. Its main characteristic is the superposition of two central fields with different strenghts of up to 2 and 0.7 V pm^−1^ near the gallium and nitrogen site, respectively. To enable the comparison with the theoretical electric field 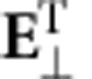 derived from the negative gradient of the Coulomb potential, 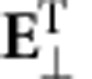 was convolved with the probe intensity according to equation (3), resulting in Fig. 2d. The excellent agreement with Fig. 2c as to magnitude and direction of the electric field demonstrates that the quantum mechanical interpretation of diffracted intensities is a key for field quantification at the atomic scale. Furthermore, we found that the integration in equation (1) can be restricted to a region slightly larger than the ronchigram (Supplementary Note 3 and Supplementary Figure 2) so that recording the central part of a diffraction pattern is sufficient in practice. In addition, scattering at phonons, plasmons and core electrons leave the above analysis unaltered for thin specimens (Supplementary Note 4 and Supplementary Figure 3).

Moreover, the divergence of the electric field is proportional to the charge density owing to Maxwell’s theory. Thus, equations (2, 3) reveal that the divergence of the momentum transfer is proportional to the charge density, convolved with the probe intensity. Figure 2e depicts div
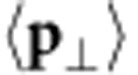
 calculated from Fig. 2b. It shows that atomic sites are sinks of momentum transfer whose magnitudes are determined by the atomic numbers. The data agree perfectly with the inset calculated from the charge density directly using density functional theory (DFT)^21^, convolved with *I*_Probe_.

The overall charge density can be expressed as 
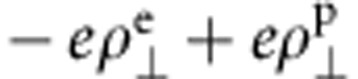
 with 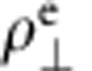 and 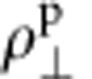 being the densities of electrons and protons, respectively. Compared with electrons, protons are spatially confined to the atomic sites that can be determined with picometre precision from the ronchigrams or from simultaneously acquired Z-contrast STEM images. Thereby, we find


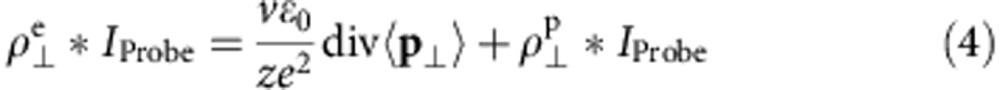


with the vacuum permittivity *ε*_0_=8.8542 × 10^−12 ^C (Vm)^−1^. This shows that the measurement of 
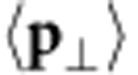
 enables mapping of the electron density convolved with the STEM probe, provided that the atomic numbers and the probe intensity are known. The latter can be recorded in practice or it can be simulated as optical parameters for probe formation are well known in aberration-corrected STEM. The proton density 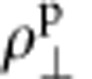 is set up by summing over Dirac’s delta functions at atom positions, weighted by the atomic numbers and eventually blurred to a Gaussian to account for thermal vibrations of the atoms. For the formal derivation of equation (4) we refer to Supplementary Note 5.

Figure 2f depicts 
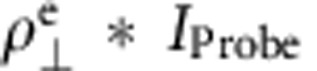
 derived from equation (4), which is obviously composed of two nearly rotationally symmetric atomic densities at the gallium and nitrogen sites, respectively. The inset shows the electron density calculated directly by DFT^21^, convolved with the STEM probe. Since nearly perfect agreement is found here, it is conceivable to measure the faint redistribution of electrons because of chemical bonding by DPC, as has been shown recently using conventional high-resolution TEM^22^.

### Experimental study of SrTiO_3_

**Figure 3:**
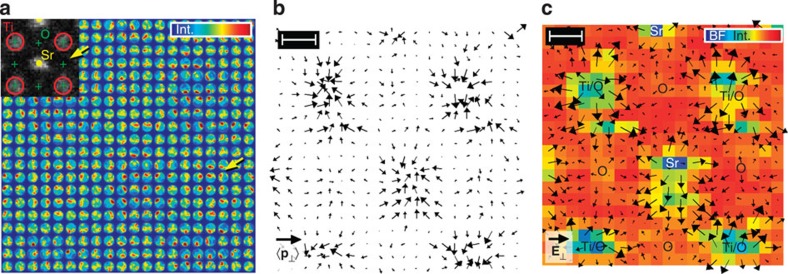
Determination of the electric field in the experimental study of SrTiO_3_. (**a**) Experimental ronchigrams of SrTiO_3_ in a region of the unit cell recorded on CCD with 4 min acquisition time. Atomic sites as indexed in the Z-contrast image (inset) are easily identified in the 20 × 20 ronchigram array. Even the oxygen columns appear clear as marked by the yellow arrow. (**b**) Experimental momentum transfer obtained from equation (1) and the ronchigrams shown in **a**. Length of the black legend vector is 5*h* nm^−1^. (**c**) Electric field 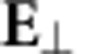 derived from the momentum transfer in **b** via equation (2) using the measured specimen thickness of *z*=2.5 nm, superimposed to the bright field intensity (BF Int.). Length of the black legend vector is 1 V pm^−1^. Scale bars top left in **b**,**c** are 100 pm.

The applicability of our approach is evidenced in an experimental case study of Strontium titanate in [100] projection, where the distance of adjacent atomic columns is a factor of 1.8 larger than in GaN. In view of experimental constraints such as specimen drift and scan noise, this material is favourable because of a large ratio of intercolumnar distance to the STEM probe diameter. An aberration-corrected STEM instrument with a semiconvergence angle of 21 mrad was employed to scan over an SrTiO_3_ unit cell region (see Methods section for details). Ronchigrams of a 20 × 20-pixel scan have been arrayed with respect to the probe positions in Fig. 3a with a STEM dark-field image and atomic sites of the perovskite unit cell as inset. By looking at the intensity redistributions inside the ronchigrams, atomic columns containing heavy atoms, such as Ti at the corners and Sr in the centre, are clearly identified by the eye at first glance. At the second glance, even pure oxygen columns emerge from the ronchigrams, as exemplified by the yellow arrows in Fig. 3a. At this stage, this experiment already confirms that atomic electric fields rather cause complex intensity variations inside the ronchigrams than global shifts.

Next we applied equation (1) to calculate the momentum transfer 
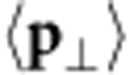
 from the ronchigrams in Fig. 3a to obtain the vector field depicted in Fig. 3b. As expected, a deflection towards atomic sites is observed clearly with a magnitude depending on the atomic numbers. In particular, atomic columns containing Sr or Ti exhibit maximum momentum transfers of ~3*h* nm^−1^, which is a factor two to three times larger compared with pure oxygen columns.

We finally proceed with the derivation of the electric field 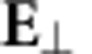 from Fig. 3b by applying equation (2). Using an atom-counting procedure via quantitative high-angle annular dark-field (HAADF) STEM analysis^23^ the specimen thickness was determined to *z*=2.5±0.4 nm. Vectors in Fig. 3c show the resulting electric field together with the colour-coded bright-field signal calculated from the ronchigrams in Fig. 3a. Obviously, all atomic columns show up as sources of the electric field with maximum field strengths of 0.4 V pm^−1^ near pure oxygen and 1 V pm^−1^ at both Ti/O and Sr columns, respectively. Owing to the extension of the STEM probe, the measured field strength decreases at the exact positions of atomic columns according to equation (3). In the following, these results are discussed with respect to the accuracy of the electric field measurement and the distortions due to scan noise (that is, random deviations of the STEM probe from a regular scan grid) obviously present in Fig. 3b,c.

**Figure 4:**
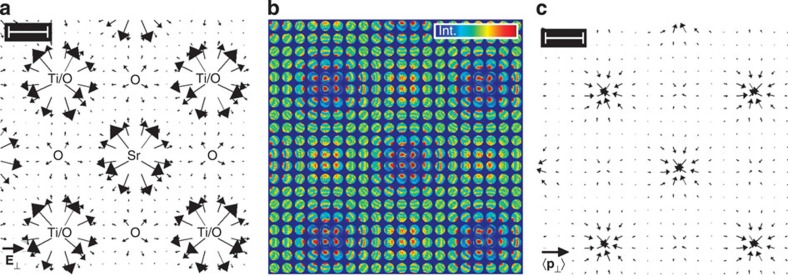
Simulations corresponding to the experimental study of SrTiO_3_. (**a**) Theoretical electric field calculated from the negative gradient of the Coulomb potential, convolved with the probe intensity according to equation (3). Length of the black legend vector is 1 V pm^−1^. (**b**) Simulation of ronchigrams at 2.5 nm specimen thickness at 20 × 20 raster positions as in the experiment in Fig. 3a. (**c**) Simulated momentum transfer calculated from simulated ronchigrams in **b** at 2.5 nm thickness. Length of the black legend vector is 5*h* nm^−1^. Scale bars top left are 100 pm. Scan noise has not been accounted for.

As to the accuracy of the measured electric field, we calculated the theoretical counterpart 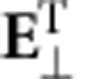 for SrTiO_3_ from the negative gradient of the Coulomb potential and convolved the result with the probe intensity as shown in Fig. 4a. Compared with the experimental field in Fig. 3c, quantitative agreement is found for the columns of pure oxygen. Essentially, this also holds for the Sr and Ti/O columns, except for very few vectors close to the respective sites where the theoretical field takes slightly larger values of up to 1.4 V pm^−1^. This suggests that a smaller sample thickness would be preferable to enhance accuracy at these positions further as we start to leave the regime of small specimen thickness, at which equation (2) is valid for atomic columns with heavy atoms, such Ti and Sr. In that case, we nevertheless expect quantitative agreement of experimental momentum transfers in Fig. 3b with momentum transfers calculated from simulated ronchigrams at 2.5 nm specimen thickness, which are arrayed in Fig. 4b. Except for scan noise, the ronchigrams exhibit tight analogy to the experimental counterpart in Fig. 3a concerning the observed redistributions of ronchigram intensities towards atomic columns. In addition, the corresponding simulated momentum transfers in Fig. 4c take maximum values of ~3*h* nm^−1^ as in the experiment of Fig. 3b.

**Figure 5:**
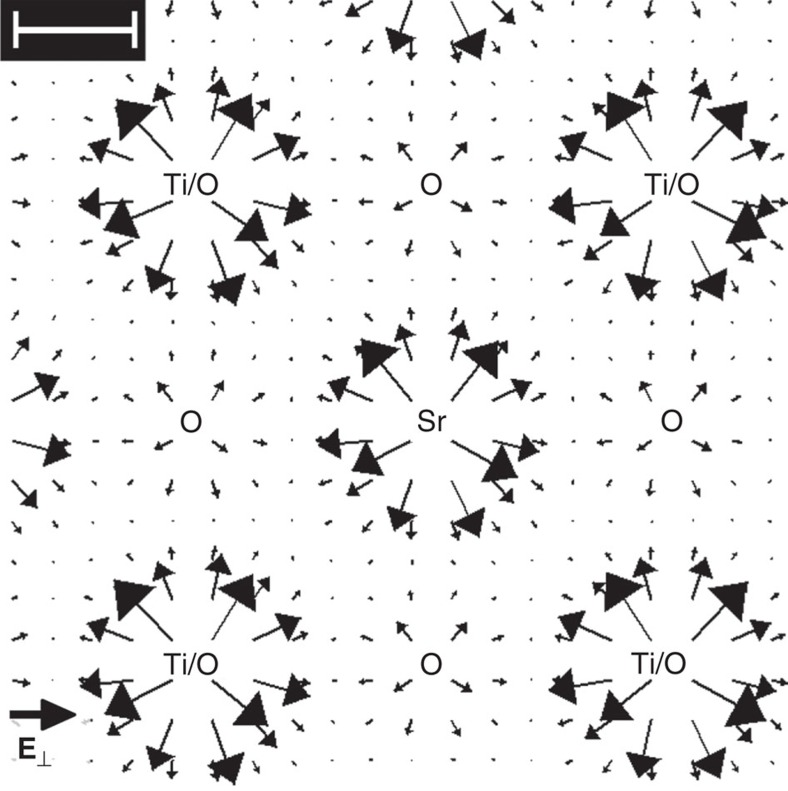
On the enhanced accuracy of electric field measurement near heavy atomic columns at lower specimen thickness. Electric field determined from simulated ronchigrams of SrTiO_3_ at 1.6 nm specimen thickness. This shows that remaining inaccuracies of the measured field in Fig. 3c in the explicit vicinity of atomic columns with heavy atoms are due to specimen thickness. To be compared directly with the theoretical result in Fig. 4a. Length of the black legend vector is 1 V pm^−1^, scale bar is 100 pm.

To complement the discussion on the influence of specimen thickness, Fig. 5 has been added, which shows the electric field that is expected for an experiment performed at slightly lower specimen thickness of 1.6 nm. In particular, ronchigrams have been simulated for that thickness, from which momentum transfer and, subsequently, electric field have been calculated via equations (1) and (2). This field is indeed practically identical with the theoretical one in Fig. 4a with a maximum error of 0.15 V pm^−1^.

**Figure 6:**
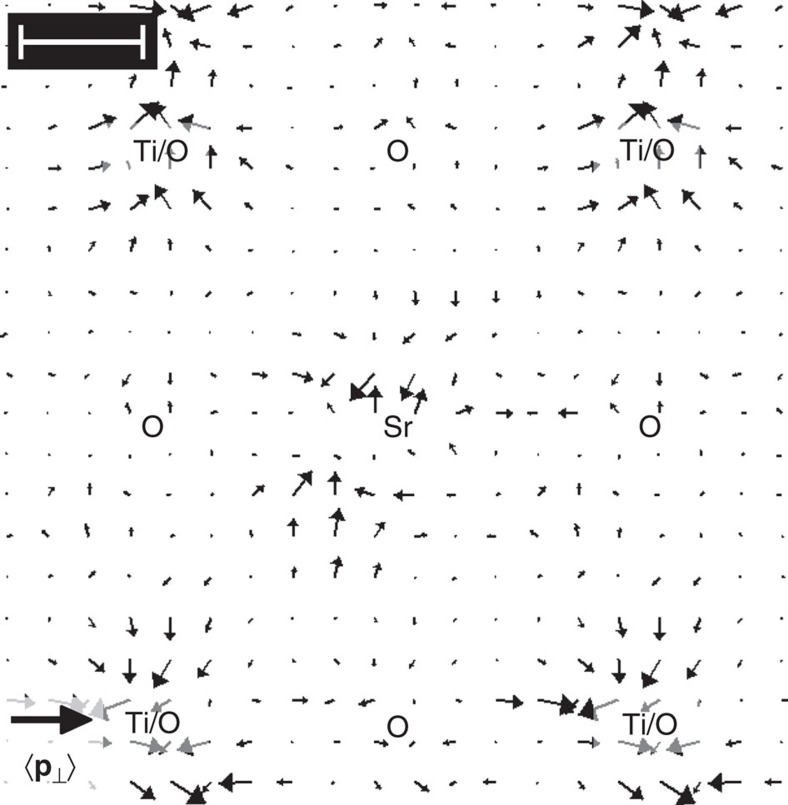
The effect of scan noise on momentum transfers in SrTiO_3_. To explain the distortions in Fig. 3b, momentum transfers have been calculated from simulated ronchigrams at 2.5-nm specimen thickness with scan noise included in the simulation (see also Methods section). Length of the black legend vector is 5*h* nm^−1^. Scale bar is 100 pm.

Moreover, momentum transfer, electric field and bright-field intensity in Fig. 3b,c are not exactly rotationally symmetric with respect to atomic sites but exhibit a slight vertical elongation and a displacement of individual scan lines in vertical and horizontal directions. This can be explained by specimen drift during the relatively long acquisition time of 4 min despite the drift correction used, and by scan noise typically present in contemporary STEM data. To verify this interpretation, SrTiO_3_ ronchigrams have been simulated with positions of the STEM probe on an irregular grid chosen appropriately to the experimental drift rate and typical scan noise parameters^24^. The resulting momentum transfers in Fig. 6 exhibit the same characteristic distortions as in Fig. 3b, which demonstrates that the observed artefacts must be attributed to residual instabilities of the instrument. Note that the magnitudes of experimental and simulated momentum transfers agree quantitatively.

**Figure 7:**
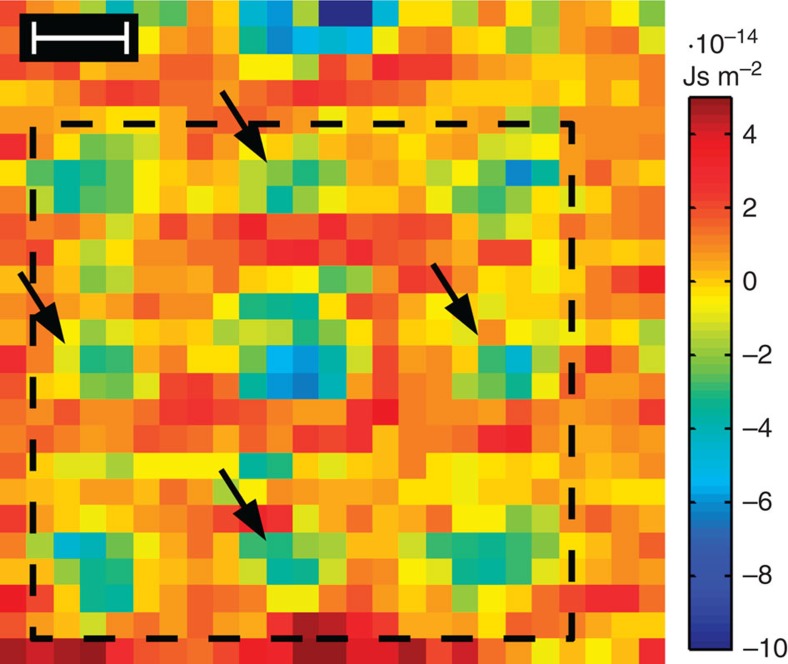
Imaging of light atoms via div 
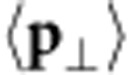
. Divergence of the vector field for the momentum transfer for a 25 × 25-pixel-large experimental scan of an SrTiO_3_ unit cell region. Atom positions emerge as significant sinks of momentum transfer. Arrows show positions of oxygen columns. Scale bar is 100 pm.

In addition, the observation that oxygen in Fig. 3b emerges significantly as a sink of momentum transfer despite its low atomic number deserves further attention, since imaging light atoms is still a major challenge in contemporary materials science^25^,^26^. As shown by the divergence of 
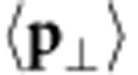
 in an SrTiO_3_ experiment with 25 × 25 scan pixels in Fig. 7, all nine atomic columns of the perovskite unit cell in [100] projection (marked by the dashed black line) are imaged with remarkably high contrast, including columns of pure oxygen indicated by arrows. Note that these columns do not show significant contrast in both the annular dark-field STEM image in Fig. 3a (inset) and the bright-field STEM image in Fig. 3c.

## Discussion

In the strict sense, Fig. 7 ought to be proportional to the charge density in analogy to the GaN counterpart in Fig. 2e. However, calculating the divergence relies on numerical differences between momenta of adjacent scan pixels, which is critical in the presence of scan noise. For this reason we restricted our interpretation to the detection of light atoms, such as oxygen. Quantitative charge- and electron density mapping will therefore profit from ongoing improvements of the stability of the scan coils of STEM instruments, or require subsequent image processing^24^. Moreover, detection quantum efficiency and readout time of current CCDs impose an upper limit of a few hundred scan points of the STEM probe to minimize drift, specimen damage and contamination. This constraint can be expected to improve drastically with the recent development of ultrafast direct electron detectors^27^ working at kHz readout rates, which are in prototype state currently. For the present, drift in the range of the STEM probe diameter during several minutes must be accepted. These experimental limitations have been pointed out already in recent ptychography work^28^, which essentially uses a similar four-dimensional data set to obtain a complex-valued object transmission function by dedicated algorithms^29^,^30^,^31^. However, ptychographic studies have as yet focused on enhancing the microscope resolution beyond the diffraction limit instead of electric field mapping so that we leave a respective methodical comparison as a future task.

The situation is different for DPC microscopy. Being a well-established approach especially for magnetic field mapping, we shall comment on its previous success despite the classical interpretation of recorded data and the current segmented detector design. For fields that are practically constant across the diameter of the STEM probe, interaction with the specimen is indeed well described by multiplication with a phase wedge, leading to a shift of the diffraction pattern as a whole without redistributions of intensity inside the ronchigram. This condition is justified for fields varying on micrometre scale as mentioned in the introduction.

To conclude, we have demonstrated how to push DPC to atomic electric field quantification in a comprehensive simulation study of GaN and both an experimental and simulation study of the important ceramics SrTiO_3_. By relating diffracted intensities to the expectation value of the momentum transfer via axioms of quantum mechanics, and furthermore converting the result to the electric field by virtue of Ehrenfest’s theorem, this constitutes a fundamentally new view on DPC experiments.

## Methods

### Simulation details

We used the multislice algorithm^17^ implemented in the StemSim software^20^ to simulate thickness-dependent diffraction patterns of GaN and SrTiO_3_ with electron beam incidences along 
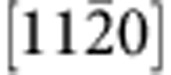
 and [100], respectively (assumed parallel to the *z* direction). This algorithm especially takes multiple scattering of electrons in the specimen into account.

For the GaN study, a contemporary aberration-corrected STEM probe with a semiconvergence angle of 21 mrad was simulated for 300 keV energy. We assumed a residual spherical aberration of *C*_s_=40 μm and a condenser operated under Scherzer conditions (giving the white intensity profile in Fig. 1c). Multislice results at a regular grid of 80 × 80 positions of the STEM probe in the red patch of Fig. 1c have been converged with respect to the lateral extension of the crystal supercell (being 15 × 15 the yellow patch in Fig. 1c), the slice thickness Δ*z* (being 0.32 nm) and the cutoff in reciprocal space (being 90 nm^−1^, corresponding to nine times the ronchigram radius). Before the multislice simulations, we used DFT as implemented in the WIEN2K^21^ software for the calculation of the electron density in GaN to include electron redistribution due to chemical bonding. The result was then converted to the 3D electrostatic Coulomb potential *V*(**r**) using the Mott–Bethe relation^32^ in the framework of the *modified atomic scattering amplitudes*^33^ model adapted for GaN. In particular, this approach is able to treat bonding in an atomistic manner that enables us to account for the thermal movement of the atoms in the framework of Debye–Waller damping. The mean squared displacements of 0.0032 and 0.0037 Å^2^ (according to 300 K) have been used for Ga and N, respectively^34^.

The SrTiO_3_ simulations have been performed analogously but using optical parameters measured experimentally as listed in Table 1 and scattering factors according to isolated atoms^35^ for both the elastic and inelastic parts. A slice thickness equal to the lattice constant of 0.3905, nm was used. The mean squared displacements of 0.01 Å^2^ for Sr, 0.0071 Å^2^ for Ti and 0.01 Å^2^ for O (according to 300 K) have been used. Scan noise in Fig. 5 was accounted for by rastering the STEM probe over an irregular grid given by random deviations from the regular positions corresponding to a Gaussian distribution with a s.d. of 20 pm. This is a rather conservative estimate as literature^24^ gives 30 pm for a typical s.d.

The 2D potential shown in Figs 1c and 2b was derived using


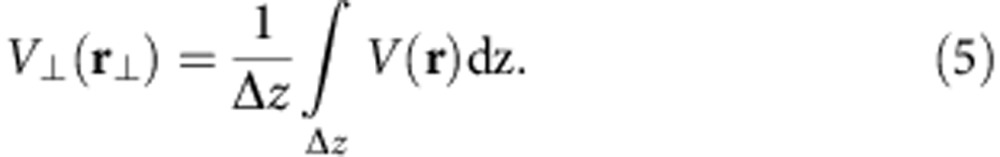


This is the Coulomb potential of one slice, averaged along the *z* direction. It is important to note that all other quantities indexed this way (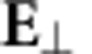, 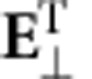, 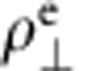, 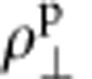) analogously refer to their average along the *z* direction.

All simulations shown in the article have been conducted in absorptive potential approximation by adding an imaginary part^35^ to the Coulomb potential derived from the mean squared displacements. This accounts for the damping of the elastic signal due to thermal diffuse scattering with increasing specimen thickness and is known to be accurate up to much higher thicknesses than considered here. To verify this, thermal diffuse scattering is explicitly treated in Supplementary Note 4.

### Experimental details

Experiments were performed at 300 kV in STEM mode on the Qu-Ant-TEM, an FEI Titan3 with probe and image aberration correctors, a monochromator and an X-FEG. The standard 50 μm condenser aperture was selected as limiting aperture with the monochromator slightly defocused to limit the beam current to ~30 pA. The SrTiO_3_ sample was thinned in the [100] direction using an FEI Helios Nanolab focused ion beam instrument with a beam energy of 30 kV. A final cleaning step was performed at low energy (2 kV) to reduce the amorphous layer thickness.

A beam semiconvergence angle of 21 mrad was used. The corrector was tuned such that the aberrations given in Table 1 have been measured.

All ronchigrams have been recorded on CCD camera with a 50-ms frame time and a sampling of 128 × 128 pixels. The SrTiO_3_ unit cell was scanned with samplings of 20 × 20 and 25 × 25 raster positions of the STEM probe because beam-induced specimen damage was observed for higher sampling rates of 30 × 30 pixels. This has been verified by comparing Z-contrast images recorded before and after acquisition of the ronchigram series, respectively. After each scan line, a drift correction has been performed by cross-correlating with a Z-contrast image of 2 × 2 SrTiO_3_ unit cells taken at 2 to 5 nm distance from the region of interest.

Specimen thickness was measured from quantitative analysis of an HAADF STEM image of the region of interest using the atom-counting procedure^23^ taking into account the response of the HAADF detector. In particular, a thickness of 5–7 unit cells (2–3 nm) was found. In addition, we checked this result by comparing the experimental annular bright-field signal with the corresponding signal extracted from simulated ronchigrams.

## Author contributions

K.M. wrote the article and did the DFT calculations. Elastic simulations and evaluations of the data have been carried out by K.M. and F.F.K. A.B. and J.V. performed the STEM experiments on SrTiO_3_ and processed the experimental data. M.S. and A.R. developed the simulation software. V.G. analysed the impact of electric field gradients. S.L. and P.S. simulated and interpreted inelastic scattering effects. The work was initiated by A.R., J.Z. and P.S. The key idea to relate DPC with the quantum mechanical momentum transfer was conceived during discussions among all authors.

## Additional information

**How to cite this article:** Müller, K. *et al.* Atomic electric fields revealed by a quantum mechanical approach to electron picodiffraction. *Nat. Commun.* 5:5653 doi: 10.1038/ncomms6653 (2014).

## Supplementary Material

Supplementary Information Supplementary Figures 1-3, Supplementary Notes 1-5 and Supplementary References.

## Figures and Tables

**Table 1:** Measured aberrations of the probe-forming system in the SrTiO_3_ experiment.

**Aberration type**	**Value**
*A*_2_ (threefold astigmatism)	55±40 nm
*B*_2_ (coma)	30±20 nm
*C*_3_ (spherical aberration C_s_)	−0.8±1.9 μm
*A*_3_ (fourfold astigmatism)	2.7±1.1 μm
*S*_3_ (star aberration)	600±650 nm

## References

[b1] Wen Z., Li C., Wu D., Li A. & Ming N. Ferroelectric-field-effect-enhanced electroresistance in metal/ferroelectric/semiconductor tunnel junctions. Nat. Mater. 12, 617–621 (2013). 23685861 10.1038/nmat3649

[b2] Tsymbal E. Y. & Kohlstedt H. Tunneling across a ferroelectric. Science 313, 181–183 (2006). 16840688 10.1126/science.1126230

[b3] Nakamura S., Mukai T. & Senoh M. Candela-class high-brightness InGaN/AlGaN double-heterostructure blue-light-emitting diodes. Appl. Phys. Lett. 64, 1687–1689 (1994).

[b4] Iso K. *et al.* High brightness blue InGaN/GaN light emitting diode on nonpolar m-plane bulk GaN substrate. Jpn. J. Appl. Phys. 46, L960–L962 (2007).

[b5] MasuiH. *et al.* Effects of piezoelectric fields on optoelectronic properties of InGaN/GaN quantum-well light-emitting diodes prepared on nonpolar 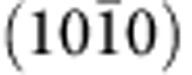 and semipolar 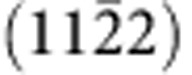 orientations. J. Phys. D. Appl. Phys. 42, 135106 (2009).

[b6] Nellist P. D. Electron microscopy: atomic resolution comes into phase. Nat. Phys. 8, 586–587 (2012).

[b7] Lohr M. *et al.* Differential phase contrast 2.0—opening new ‘‘fields'' for an established technique. Ultramicroscopy 117, 7–14 (2012). 22634135 10.1016/j.ultramic.2012.03.020

[b8] Shibata N. *et al.* Differential phase-contrast microscopy at atomic resolution. Nat. Phys. 8, 611–615 (2012).

[b9] Tsymbal E. Y. & Gruverman A. Ferroelectric tunnel junctions: beyond the barrier. Nat. Mater. 12, 602–604 (2013). 23685862 10.1038/nmat3669

[b10] Takeuchi T. *et al.* Quantum-confined stark effect due to piezoelectric fields in GaInN strained quantum wells. Jpn. J. Appl. Phys. 36, L382–L385 (1997).

[b11] Ploessl R., Chapman J. N., Thompson A. M., Zweck J. & Hoffmann H. Investigation of the micromagnetic structure of cross-tie walls in permalloy. J. Appl. Phys. 73, 2447–2452 (1993).

[b12] Zweck J., Zimmermann T. & Schuhrke T. TEM imaging and evalution of magnetic structures in Co/Cu multilayers. Ultramicroscopy 67, 153–162 (1997).

[b13] Sandweg C. W. *et al.* Direct observation of domain wall structures in curved permalloy wires containing an antinotch. J. Appl. Phys. 103, 093906 (2008).

[b14] Rose H. Nonstandard imaging methods in electron microscopy. Ultramicroscopy 2, 251–267 (1977). 888244 10.1016/s0304-3991(76)91538-2

[b15] Chapman J., Batson P., Waddell E. & Ferrier R. The direct determination of magnetic domain wall profiles by differential phase contrast electron microscopy. Ultramicroscopy 3, 203–214 (1978). 358526 10.1016/s0304-3991(78)80027-8

[b16] Shibata N. *et al.* New area detector for atomic-resolution scanning transmission electron microscopy. J. Electron Microsc. (Tokyo) 59, 473–479 (2010). 20406732 10.1093/jmicro/dfq014

[b17] Cowley J. M. & Moodie A. F. The scattering of electrons by atoms and crystals. I. A new theoretical approach. Acta Crystallogr. 10, 609–619 (1957).

[b18] Bethe H. Theorie der Beugung von Elektronen an Kristallen. Ann. Phys. 87, 55–129 (1928).

[b19] Ehrenfest P. Bemerkung über die angenäherte Gültigkeit der klassischen Mechanik innerhalb der Quantenmechanik. Z. Phys. A 45, 455–457 (1927).

[b20] Rosenauer A. & Schowalter M. InSpringer Proceedings in Physics vol. 120, (eds Cullis A. G., Midgley P. A. 169–172Springer (2007).

[b21] Blaha P., Schwarz K., Madsen G., Kvasnicka D. & Luitz J. Wien2k, An Augmented Plane Wave+Local Orbitals Program for Calculating Crystal Properties Technische Universität Wien: Austria, (2001).

[b22] Meyer J. C. *et al.* Experimental analysis of charge redistribution due to chemical bonding by high-resolution transmission electron microscopy. Nat. Mater. 10, 209–215 (2011). 21240288 10.1038/nmat2941

[b23] Van Aert S., Batenburg K. J., Rossell M. D., Erni R. & Van Tendeloo G. Three-dimensional atomic imaging of crystalline nanoparticles. Nature 470, 374–377 (2011). 21289625 10.1038/nature09741

[b24] Jones L. & Nellist P. D. Identifying and correcting scan noise and drift in the scanning transmission electron microscope. Microsc. Microanal. 19, 1050–1060 (2013). 23673234 10.1017/S1431927613001402

[b25] Jia C. L. & Urban K. Atomic-resolution measurement of oxygen concentration in oxide materials. Science 303, 2001–2004 (2004). 15044799 10.1126/science.1093617

[b26] Findlay S. D. *et al.* Dynamics of annular bright field imaging in scanning transmission electron microscopy. Ultramicroscopy 110, 903–923 (2010). 20434265 10.1016/j.ultramic.2010.04.004

[b27] Müller K. *et al.* Scanning transmission electron microscopy strain measurement from millisecond frames of a direct electron charge coupled device. Appl. Phys. Lett. 101, 212110 (2012).

[b28] Humphry M. J., Kraus B., Hurst A. C., Maiden A. M. & Rodenburg J. M. Ptychographic electron microscopy using high-angle dark-field scattering for sub-nanometre resolution imaging. Nat. Commun. 3, 730 (2011). 22395621 10.1038/ncomms1733 PMC3316878

[b29] Rodenburg J. M. & Bates R. H. T. The theory of super-resolution electron microscopy via Wigner-distribution deconvolution. Phil. Trans. R Soc. A 339, 521–553 (1992).

[b30] Rodenburg J. M., McCallum B. C. & Nellist P. D. Experimental tests on double-resolution coherent imaging via STEM. Ultramicroscopy 48, 304–314 (1993).

[b31] Maiden A. M. & Rodenburg J. M. An improved ptychographical phase retrieval algorithm for diffractive imaging. Ultramicroscopy 109, 1256–1262 (2009). 19541420 10.1016/j.ultramic.2009.05.012

[b32] Mott N. F. The scattering of electrons by atoms. Proc. R. Soc. Lond. A 127, 658–665 (1930).

[b33] Rosenauer A., Schowalter M., Glas F. & Lamoen D. First-principles calculations of 002 structure factors for electron scattering in strained In_x_Ga_1−x_As. Phys. Rev. B 72, 085326 (2005).

[b34] Schowalter M., Rosenauer A., Titantah J. & Lamoen D. Temperature-dependent Debye-Waller factors for semiconductors with the wurtzite-type structure. Acta Crystallogr. A 65, 227–231 (2009). 19349666 10.1107/S0108767309004966

[b35] Weickenmeier A. & Kohl H. Computation of absorptive form factors for high-energy electron diffraction. Acta Crystallogr. A 47, 590–597 (1991).

