# Supplementary material for: Atomic electric fields revealed by a quantum mechanical approach to electron picodiffraction
Source: Nat Commun. 2014 Dec 15;5:5653. doi: 10.1038/ncomms6653 (PMC4275586; doi:10.1038/ncomms6653)
Supplement: Supplementary Information — Supplementary Figures 1-3, Supplementary Notes 1-5 and Supplementary References. [file ncomms6653-s1.pdf]

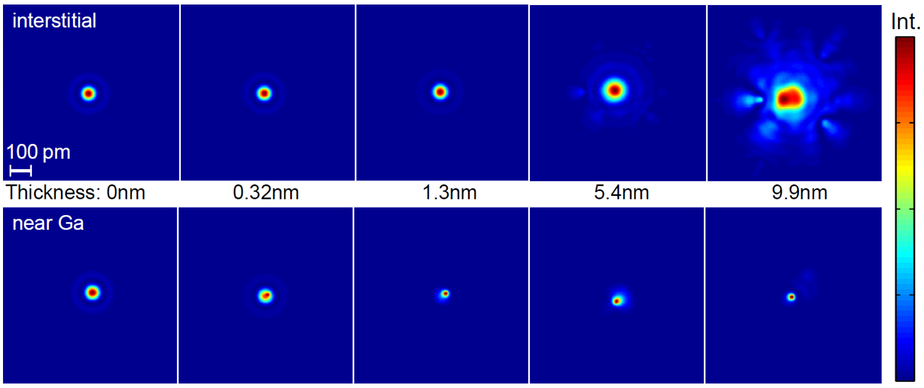

Supplementary Figure 1: Investigation of the broadening of the STEM probe with increasing specimen thickness at positions 1 and 2 in Fig. 1 c. The maps depict the intensity of the electron wave inside the specimen for the indicated thicknesses, showing that intensity remains confined to the region of the incident probe (leftmost graphics) at least for thicknesses up to 1.3 nm. Severe broadening is found beyond 5 nm thickness, especially in the interstitial region. The STEM probe has a semiconvergence angle of 21 mrad in this simulation.

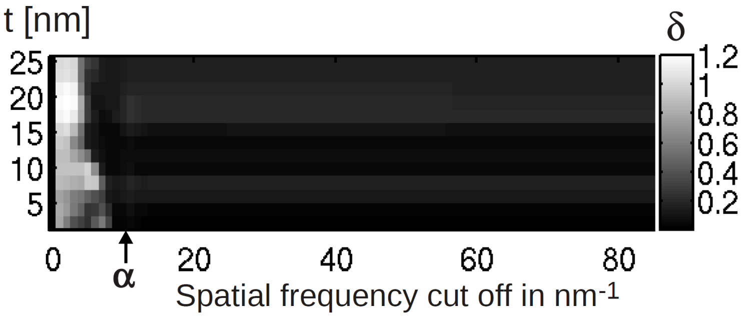

Supplementary Figure 2: Comparison of directions of the electric field determined from momentum transfers and those of the true field. Fields analogous to Fig. 2 c have been calculated in dependence of spatial frequency cutoff in equation (1) and specimen thickness t. The gray scale shows the directional error δ defined in the text. Nearly exact agreement ($\delta \approx 0$) is found for cutoffs larger than the ronchigram radius α.

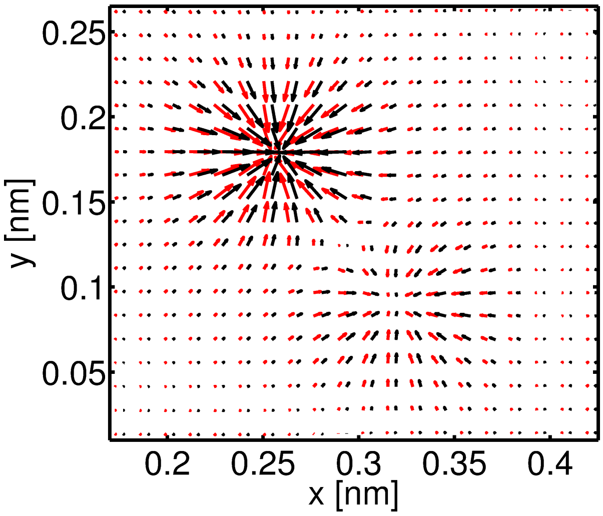

Supplementary Figure 3: The influence thermal diffuse scattering (TDS) on the momentum transfer in GaN. The black field was calculated from diffraction patterns simulated with absorptive potentials where TDS does not show up explicitly in the diffraction pattern. The red one (shifted slightly to facilitate comparison) stems from a frozen phonon simulation with 120 thermal configurations and explicitly includes TDS. Both fields are practically identical.

Supplementary Note 1

Broadening of the STEM probe inside the specimen

In conjunction with equation (2) we stated that a specimen thickness of 1-2 nm is low enough to neglect broadening of the STEM probe which we verify now. To this end, the intensity of the electron wave was simulated thickness dependent as described in the Methods section for probe positions 1 and 2 in Fig. 1 c, being representative for interstitial regions and regions with high electric fields. For selected thicknesses, the result is depicted in Supplementary Figure 1. For both the top and bottom row we see that broadening is in fact negligible at 1.3 nm (considered in the article) and significantly rises beyond 5 nm. Note that broadening is an issue mostly in the interstitial region since there is no electric field that confines the divergent STEM probe spatially, opposite to the Ga site where electrons preferably channel along atom columns and are even focused with increasing thickness.

Supplementary Note 2

Justification of equations (1)-(3)

It is one of the fundamental axioms of quantum mechanics that the expectation value of an observable represented by an operator A, given a state $|\psi\rangle$ is

$\langle A \rangle = \langle \psi | A | \psi \rangle$. Thus, the expectation value of the momentum operator $\mathbf{p}_\perp$ (in momentum space representation) in a certain plane is given by

$$\begin{aligned}\langle \mathbf{p}_\perp \rangle &= \langle \psi | \mathbf{p}_\perp | \psi \rangle \\ &= \int \mathrm{d}^2 p_\perp \mathbf{p}_\perp \psi(\mathbf{p}_\perp)^* \psi(\mathbf{p}_\perp) \\ &= \int \mathrm{d}^2 p_\perp \mathbf{p}_\perp I(\mathbf{p}_\perp).\end{aligned} \tag{1}$$

This is equation (1).

Equivalently, we obtain in real space representation:

$$\begin{aligned}\langle \mathbf{p}_\perp \rangle &= -\mathrm{i}\hbar \int \mathrm{d}^2 r_\perp \psi(\mathbf{r}_\perp)^* \nabla \psi(\mathbf{r}_\perp) \\ &= -\mathrm{i}\hbar \int \mathrm{d}^2 r_\perp \int \mathrm{d}^2 p_\perp \exp(-\mathrm{i}\hbar^{-1} \mathbf{p}_\perp \mathbf{r}_\perp) \psi(\mathbf{p}_\perp)^* \\ &\quad \times \nabla \int \mathrm{d}^2 p' \exp(\mathrm{i}\hbar^{-1} \mathbf{p}'_\perp \mathbf{r}_\perp) \psi(\mathbf{p}'_\perp) \\ &= -\mathrm{i}^2 \hbar \hbar^{-1} \int \mathrm{d}^2 p_\perp \int \mathrm{d}^2 p'_\perp \psi(\mathbf{p}_\perp)^* p'_\perp \psi(\mathbf{p}'_\perp) \\ &\quad \times \underbrace{\int \mathrm{d}^2 r_\perp \exp(\mathrm{i}\hbar^{-1} (\mathbf{p}'_\perp - \mathbf{p}_\perp) \mathbf{r}_\perp)}_{=\delta(\mathbf{p}_\perp - \mathbf{p}'_\perp)} \\ &= \int \mathrm{d}^2 p_\perp \psi(\mathbf{p}_\perp)^* p_\perp \psi(\mathbf{p}_\perp).\end{aligned} \tag{2}$$

Here, it was exploitet that momentum- and real space representation are related by Fourier transform. The result is obviously also identical to equation (1), which is not surprising as expectation values cannot depend on representation.

This expectation value is, of course, dependent on the wave function and the plane in which it is evaluated, i.e., the value of z. To relate different z values, we use the Ehrenfest theorem[1]:

$$\frac{\mathrm{d}}{\mathrm{d}t} \langle A \rangle = \frac{1}{\mathrm{i}\hbar} \langle [A, H] \rangle + \left\langle \frac{\partial A}{\partial t} \right\rangle \tag{3}$$

where A is again an arbitrary operator and H is the system's Hamiltonian. Applying this theorem to the momentum operator $\mathbf{p}_\perp$ in the Schrödinger picture (where $\partial \mathbf{p}_\perp / \partial t = 0$) gives

$$\frac{\mathrm{d}}{\mathrm{d}t} \langle \mathbf{p}_\perp \rangle = \frac{1}{\mathrm{i}\hbar} \langle [\mathbf{p}_\perp, H] \rangle . \tag{4}$$

With $H = \frac{p^2}{2m_\mathrm{e}} - eV$ and $\mathbf{p}_\perp = -\mathrm{i}\hbar \nabla_\perp$ we get

$$[\mathbf{p}_\perp, p^2] = 0 \quad \text{and} \quad [\mathbf{p}_\perp, V] = -\mathrm{i}\hbar \, \mathrm{grad}_\perp \, V = \mathrm{i}\hbar \mathbf{E}_\perp^{\mathrm{full}}. \tag{5}$$

where p is the momentum operator, m_e and e are the mass and charge of the electron and V is the electrostatic potential inside the specimen. The superscript "full" of $\mathbf{E}_\perp^\mathrm{full}$ indicates that this is the lateral component of the still full three-dimensional field as opposed to the z-averaged fields that are used in the main article. Collecting these results yields

$$\begin{aligned} \frac{\mathrm{d}}{\mathrm{d}t} \langle \mathbf{p}_\perp \rangle &= \frac{1}{\mathrm{i}\hbar} \langle [\mathbf{p}_\perp, H] \rangle \\ &= -e \left\langle \mathbf{E}_\perp^\mathrm{full} \right\rangle \quad . \end{aligned} \tag{6}$$

Until here we considered non-relativistic cases only. In a slighly lengthier calculation which starts from the Dirac equation and hence takes relativistic effects into account correctly, one obtains for the general case with vector potential $\mathbf{A}$ and electric field $\mathbf{E}^\mathrm{full}$

$$\frac{\mathrm{d}}{\mathrm{d}t} \langle \mathbf{p}_\perp + e\mathbf{A} \rangle = -e \left\langle \mathbf{v} \times \mathrm{rot}\mathbf{A} + \mathbf{E}_\perp^\mathrm{full} \right\rangle \tag{7}$$

with the Lorentz force on the right. This is equivalent to the non-relativistic case, and with vanishing magnetic field ($\mathbf{A} = 0$) we obtain the above result.

With $\frac{\mathrm{d}}{\mathrm{d}t} = \frac{\mathrm{d}z}{\mathrm{d}t}\frac{\mathrm{d}}{\mathrm{d}z} = v\frac{\mathrm{d}}{\mathrm{d}z}$ in the paraxial approximation, $\left\langle \mathbf{E}_\perp^\mathrm{full} \right\rangle$ is a function of z and we obtain

$$\frac{\mathrm{d}}{\mathrm{d}z} \langle \mathbf{p}_\perp \rangle = -\frac{e}{v} \left\langle \mathbf{E}_\perp^\mathrm{full} \right\rangle . \tag{8}$$

The expectation value $\left\langle \mathbf{E}_\perp^\mathrm{full} \right\rangle = \left\langle \psi | \mathbf{E}_\perp^\mathrm{full} | \psi \right\rangle$ is defined as the integral

$$\left\langle \mathbf{E}_\perp^\mathrm{full} \right\rangle = \int \mathrm{d}^2 r_\perp \psi^*(\mathbf{r}_\perp, z) \mathbf{E}_\perp^\mathrm{full}(\mathbf{r}_\perp, z) \psi(\mathbf{r}_\perp, z) \tag{9}$$

with $\psi(\mathbf{r}_\perp, z)$ the wave function inside the specimen depending on $\mathbf{r}_\perp = (x, y)$ and z. This is equivalent to

$$\left\langle \mathbf{E}_\perp^\mathrm{full} \right\rangle = \int \mathrm{d}^2 r_\perp \psi^*(\mathbf{r}_\perp, z) \psi(\mathbf{r}_\perp, z) \mathbf{E}_\perp^\mathrm{full}(\mathbf{r}_\perp, z) = \int \mathrm{d}^2 r_\perp I(\mathbf{r}_\perp, z) \mathbf{E}_\perp^\mathrm{full}(\mathbf{r}_\perp, z) \quad . \tag{10}$$

In the second step, we used the fact that $\psi^*(\mathbf{r}_\perp, z)\psi(\mathbf{r}_\perp, z)$ equals the intensity $I(\mathbf{r}_\perp, z)$. Note that this is the *local* intensity inside the specimen, which depends on x, y and z. To calculate it, the local wave function $\psi(\mathbf{r}_\perp, z)$ would have to be known, for example by propagating the wave function of the incident electron probe through the specimen using, e.g., the multislice algorithm. However, since in the experiment $\psi(\mathbf{r}_\perp, z)$ is not known in general (even for an exactly defined input wave it depends on the unknown electric field), an approximation for $I(\mathbf{r}_\perp, z)$ is made below.

By integration along z the total change of the average momentum reads

$$\Delta \langle \mathbf{p}_\perp \rangle = -\frac{e}{v} \int \mathrm{d}z \left\langle \mathbf{E}_\perp^\mathrm{full} \right\rangle = -\frac{e}{v} \iint \mathrm{d}^2 r_\perp \mathrm{d}z \mathbf{E}_\perp^\mathrm{full}(\mathbf{r}_\perp, z) I(\mathbf{r}_\perp, z) \tag{11}$$

with appropriate integration boundaries. Note that Supplementary Equation (11) was derived from general quantum-mechanical statements.

To be able to perform the integration, we use the simplified model that the intensity is independent of z (which is approximately the case for thin specimens considered here) and hence we have $I(\mathbf{r}_\perp, z) \approx I(\mathbf{r}_\perp, z=0)$ with $I(\mathbf{r}_\perp, z=0)$ the intensity at the specimen entrance surface. We now define an electric field averaged in z-direction as

$$\mathbf{E}_\perp^{\mathrm{T}}(\mathbf{r}_\perp) = \frac{1}{\Delta z}\int_{\Delta z} \mathrm{d}z \mathbf{E}_\perp^{\mathrm{full}}(\mathbf{r}_\perp, z). \tag{12}$$

Setting the coordinate system such that $\langle \mathbf{p}_\perp \rangle = 0$ at $z = 0$ the change of momentum becomes

$$\langle \mathbf{p}_\perp \rangle = -\frac{ez}{v}\int \mathrm{d}^2 r_\perp \mathbf{E}_\perp^{\mathrm{T}}(\mathbf{r}_\perp) I(\mathbf{r}_\perp, z=0). \tag{13}$$

As the STEM probe always has the same intensity profile only shifted by the probe position $\mathbf{R}_\perp$, we have $I(\mathbf{r}_\perp, z=0) = I_{\mathrm{Probe}}(\mathbf{R}_\perp - \mathbf{r}_\perp)$. Hence the integral is identified as a convolution which we abbreviate by $\mathbf{E}_\perp(\mathbf{R}_\perp)$:

$$\mathbf{E}_\perp(\mathbf{R}_\perp) = \int \mathrm{d}^2 r_\perp \mathbf{E}_\perp^{\mathrm{T}}(\mathbf{r}_\perp) I_{\mathrm{Probe}}(\mathbf{R}_\perp - \mathbf{r}_\perp) = \mathbf{E}_\perp^{\mathrm{T}} \otimes I_{\mathrm{Probe}}, \tag{14}$$

which is equation (3). After rearrangement, this results in equation (2):
$\mathbf{E}_\perp = -\frac{v}{ez}\langle \mathbf{p}_\perp \rangle$.

Note that the only assumption above is that the intensity distribution of the STEM probe does not change as a function of z. The interaction with the specimen, as well as the propagation of the electron wave itself, make this assumption valid for small specimen thicknesses. As to conserving the shape of the probe intensity as a function of z during propagation, curved phase fronts already of the incident STEM probe (at $z = 0$) can have a negative effect which is why we mention alignment aspects in conjunction with equation (2) in the main article. However, within this simplified model where propagation is neglected, this result shows that there is no formal restriction to flat wave fronts at $z = 0$.
See also Supplementary Note 1 as well as Fig.2 c,d (GaN), and Fig. 5 ($SrTiO_3$) where the reliability of the above simplifications is verified for the thickness range considered here via multislice simulations.

Supplementary Note 3

On the integration limits for momentum transfer measurements
In the strict sense, the expectation value for the momentum transfer must be derived from the integration over the whole momentum space according to equation (1) which is impossible in practice. All results presented for GaN have been derived by integration up to spatial frequencies of $90\,\mathrm{nm}^{-1}$ which is justified as we found that all electrons are scattered safely inside this area. As to experiments, it is interesting to study if the integral in equation (1) converges at a lower spatial frequency in order to keep the recording pixel array small enough.

We studied this convergence by calculating momentum transfers as in Fig. 2 b as a function of the spatial frequency cutoff for the integration in equation (1). All momentum transfers obtained this way have been converted to electric fields in complete analogy to Fig. 2 c using equation (2). Finally, we compared these electric fields with their theoretical counterparts as shown in Fig. 2 d. The result was that the agreement of both did not change if cutoffs slightly larger than the ronchigram radius have been used.

This analysis is exemplified for the agreement of the orientations of both fields in Supplementary Figure 2 which can be understood as follows. The quantity shown in grayscale is defined as $\delta := \overline{|1-\cos\theta|}$. For example, θ is the angle between one electric field vector in Fig. 2 c and its theoretical counterpart in Fig. 2 d. $|1-\cos\theta|$ is hence zero if these vectors are parallel and is thus a measure of the misorientation between both fields. To present results in a compact manner, δ is defined as an average over all misorientations in, e.g., the field in Fig. 2 c. This analysis has been performed as a function of specimen thickness and spatial frequency cutoff, giving δ as shown in Supplementary Figure 2. As most of the map is black which corresponds to $\delta = 0$, the directions of the electric field determined from the momentum transfer is correct for nearly all spatial frequency cutoffs beyond the radius of the ronchigram indicated by α. Moreover, this holds for very high specimen thicknesses up to 25 nm. However, analogous analysis of the error for the electric field *magnitude* revealed that quantification fails for thicknesses above 5 nm. In any case restricting the integration to the interiour of the ronchigram will result in large errors, for example misorientations larger than $\pi/2$ occur where $\delta > 1$ in Supplementary Figure 2. In particular, conventional DPC detectors as in Fig. 1 b are not suitable to put this integration into practice.

Supplementary Note 4

Influence of thermal diffuse scattering (TDS). TDS typically causes a diffuse, smooth background intensity in electron diffraction patterns which usually gains importance at higher specimen thickness. Nevertheless we checked the contribution of TDS using the frozen phonon approach[5] with an average over 120 thermal displacement configurations according to the mean squared displacements for 300 K given in the Methods section for GaN. Isolated atom scattering factors[6] have been used to set up the Coulomb potential of the crystal to avoid a computationally demanding DFT simulation for each thermal configuration. The frozen phonon result for the momentum transfer at 1.3 nm specimen thickness is compared with its counterpart derived from absorptive potential simulations in Supplementary Figure 3. Both vector fields are in excellent agreement with a relative error well below 2%. Hence TDS does not alter the findings of this article. Note that even significant amounts of TDS only affect the momentum transfer if it distributes acentrically.

Influence of plasmon and core losses. The strongest inelastic contribution in both metals and semiconductors emanates from plasmons. Although the plasma resonance itself is strongly localised in metals with a correlation length

of the order of a few Å only, the plasmon *signal* in STEM is delocalised to several nanometers owing to the long-range Coulomb interaction between probe and target[7]. This delocalisation corresponds to a very narrow Lorentzian angular distribution for plasmon scattering and is a general characteristic of all low losses. An obvious consequence is that the inelastically scattered probe continues to propagate in the same direction as before the inelastic scattering process, with tiny deviations of the order of 0.05 mrad for 300 keV primary energy. This leads to the preservation of diffraction contrast in the energy loss signal. By the same token it is responsible for the lattice periodic contrast in plasmon filtered images. We can therefore anticipate that the contribution of the plasmon losses will not alter the elastic ronchigram significantly. To verify this, diffraction patterns of plasmon losses have been calculated, based on the model of an isotropic dynamic form factor in dipole approximation[7,8]. Due to the high delocalisation of low loss scattering, we replaced the continuously distributed scattering centers in the medium by kernels located at the atom positions. This reduces the numerical effort to a manageable amount and still mimics the salient features of low loss scattering. Up to numerical accuracy, the ronchigrams are indeed found to be identical to those depicted in Fig. 2 a, in perfect agreement with the assumption above. Hence, plasmon scattering does not alter the momentum transfers derived from the ronchigrams in the thickness regime relevant for DPC.
In terms of intensity, core-loss scattering is typically several orders of magnitude weaker than low-loss scattering. As such, its influence on the ronchigrams is negligible.

Supplementary Note 5

From momentum transfer to electron density
Inserting equation (2) in equation (3) yields

$$\langle \mathbf{p}_\perp \rangle = -\frac{ez}{v}\mathbf{E}_\perp^{\mathrm{T}} \otimes I_{\mathrm{Probe}} \tag{15}$$

for small specimen thicknesses. Note that, according to the STEM geometry, only components perpendicular to the incident electron beam are accessible (index $\perp$) whereas the z-dependence is replaced by the average along z-direction. The divergence of Supplementary Equation (15) reads

$$\mathrm{div}_\perp \langle \mathbf{p}_\perp \rangle = -\frac{ez}{v}\mathrm{div}_\perp \left[\mathbf{E}_\perp^{\mathrm{T}}\right] \otimes I(\mathbf{r}_\perp) \tag{16}$$

after permuting differentiation and integration and recognizing that the divergence acts on $\mathbf{E}_\perp^{\mathrm{T}}$ only in the convolution integral.

Furthermore, $\varepsilon_0\,\mathrm{div}_\perp \mathbf{E}_\perp^{\mathrm{T}}$ is equal to the (projected) charge density owing to Maxwell's equations. Expressing the overall charge density in terms of electron and proton densities $\rho_\perp^{\mathrm{e}}$ and $\rho_\perp^{\mathrm{p}}$, respectively, and inserting above yields

$$\mathrm{div}_\perp \langle \mathbf{p}_\perp \rangle = -\frac{e^2 z}{v\varepsilon_0}\left[-\rho_\perp^{\mathrm{e}} + \rho_\perp^{\mathrm{p}}\right] \otimes I_{\mathrm{Probe}} \quad . \tag{17}$$

Solving for the electron density convolved with the STEM probe gives equation (4).

To check the reliability of equation (4), $\rho_\perp^{\mathrm{e}}$ was calculated from DFT[4] while $\rho_\perp^{\mathrm{p}}$ was set up as a summation in Fourier space

$$\rho_\perp^{\mathrm{p}}(\mathbf{r}_\perp) = \mathcal{F}^{-1}\left[\sum_{j=1}^{N} Z_j D_j(q_\perp)\,\mathrm{e}^{2\pi i \mathbf{q}_\perp \mathbf{r}_{j,\perp}}\right] \tag{18}$$

with $\mathbf{q}_\perp$ a reciprocal space vector perpendicular to z-direction. In Supplementary Equation (18), j indexes an atom with atomic number Z_j and Debye-Waller factor D_j at position $\mathbf{r}_{j,\perp}$. In our case, the summation ran over all $N = 1800$ atoms in one slice used in the multislice[2,3] simulation. For deriving the main part of Fig. 2 f, Supplementary Equation (18) was inserted in equation (4) as prior knowledge. The inset was calculated by insertion to the expression for $\mathrm{div}_\perp\langle\mathbf{p}_\perp\rangle$ above.

Supplementary References

[1] Ehrenfest, P. Bemerkung über die angenäherte Gültigkeit der klassischen Mechanik innerhalb der Quantenmechanik. *Z. Phys. A* **45**, 455–457 (1927).

[2] Cowley, J. M. & Moodie, A. F. The scattering of electrons by atoms and crystals. I. A new theoretical approach. *Acta Crystallogr.* **10**, 609–619 (1957).

[3] Rosenauer, A. & Schowalter, M. STEMSIM-a new software tool for simulation of STEM HAADF Z-contrast imaging. In Cullis, A. G. & Midgley, P. A. (eds.) *Springer Proceedings in Physics*, vol. 120, 169–172 (Springer, 2007).

[4] Blaha, P., Schwarz, K. & G. K. H. Madsen, J. L., D. Kvasnicka. *Wien2k, An Augmented Plane Wave + Local Orbitals Program for calculating crystal properties.* ISBN 3-9501031-1-2 (Technische Universität Wien, Austria, 2001).

[5] van Dyck, D. Is the frozen phonon model adequate to describe inelastic phonon scattering? *Ultramicroscopy* **109**, 677–682 (2009).

[6] Weickenmeier, A. & Kohl, H. Computation of absorptive form factors for high-energy electron diffraction. *Acta Crystallogr., Sect. A* **47**, 590–597 (1991).

[7] Schattschneider, P. & Lichte, H. Correlation and the density-matrix approach to inelastic electron holography in solid state plasmas. *Phys. Rev. B* **71**, 045130 (2005).

[8] Schattschneider, P., Nelhiebel, M. & Jouffrey, B. Density matrix of inelastically scattered fast electrons. *Phys. Rev. B* **59**, 10959–10969 (1999).
